# Industrial Internet-enabled Resilient Manufacturing Strategy in the Wake of COVID-19 Pandemic: A Conceptual Framework and Implementations in China

**DOI:** 10.1186/s10033-021-00573-4

**Published:** 2021-05-31

**Authors:** Tao Peng, Qiqi He, Zheng Zhang, Baicun Wang, Xun Xu

**Affiliations:** 1 State Key Laboratory of Fluid Power and Mechatronic Systems, Zhejiang University, Hangzhou, 310027 China; 2 Institute of Industrial Engineering, School of Mechanical Engineering, Zhejiang University, Hangzhou, 310027 China; 3 Department of Mechanical Engineering, School of Engineering, The University of Auckland, Auckland, 1010 New Zealand

**Keywords:** Resilient manufacturing, Industrial Internet, COVID-19 pandemic, Manufacturing strategy

## Abstract

COVID-19 pandemic has accelerated the re-shaping of globalized manufacturing industry. Achieving a high level of resilience is thereby a recognized, essential ability of future manufacturing systems with the advances in smart manufacturing and Industry 4.0. In this work, a conceptual framework for resilient manufacturing strategy enabled by Industrial Internet is proposed. It is elaborated as a four-phase, closed-loop process that centered on proactive industry assessment. Key enabling technologies for the proposed framework are outlined in data acquisition and management, big data analysis, intelligent services, and others. Industrial Internet-enabled implementations in China in response to COVID-19 have then been reviewed and discussed from 3Rs’ perspective, i.e. manufacturer capacity Recovery, supply chain Resilience and emergency Response. It is suggested that an industry-specific and comprehensive selection coordinated with the guiding policy and supporting regulations should be performed at the national, at least regional level.

## Introduction

In 2020, the world had been seriously struck by the outbreak of COVID-19 pandemic. Such a disaster leads to severe damage, even fracture, to the supply chain in many industries, due to the city-to-country level lockdown and travel suspension. This not only harms the globalized manufacturing network from a supply perspective, but also extremely changes consumers’ demand structure, and consequently hampers economic growth [1]. Specifically, the production of medical supplies, e.g., facemasks, protective clothing, thermometers, ventilator, and extracorporeal membrane oxygenation (ECMO), were in short supply, meanwhile, consumer market was flagging. Several automotive and shipbuilding manufacturers have restructured or reconfigured production lines and corresponding supply chains to convert their production capacity for emergency supplies [2].

Governments around the world did their utmost to help manufacturing industry pull through, including emergency deployment, financial subsidy. Besides, uncertain factors in the international situation, e.g., geopolitics, urge all governments to rebuild an independent, controllable, reliable and effective industrial chain. The newly established systematic “dual circulation” development pattern of China is looking at the domestic market as the country’s economic mainstay with domestic and foreign markets complementing each other [3]. This further raises the bar for manufacturing systems in terms of responsiveness, stability and resilience, with the advances in smart manufacturing and Industry 4.0.

Resilience, originates from psychology and ecology [4], represents the ability of a system to resist unexpected impacts, deform, and adaptively recover. In supply chain fields, design and achieve resilience has been a hot topic for decades [5]. Ivanov et al. [6] has conducted a series of studies on how to integrate agility in production capacity conversion, resilience and viability in supply chain. Existing manufacturing paradigms, such as agile manufacturing, reconfigurable manufacturing, and cloud manufacturing, aim to meet dynamic and personalized demand in a cost-effective manner, by manufacturing resource sharing and optimized configuration. However, their intentions were not to cope with severe, unexpected impacts.

Industrial Internet, as one of the fastest-growing technologies, has demonstrated its potentials and played an important role in supporting manufacturing and supply chain recovery [7]. To this end, a conceptual framework for Industrial Internet-enabled resilient manufacturing strategy is proposed in this work. Resilience in smart manufacturing is defined as the ability to (1) proactively assess and prepare before the unexpected impacts, (2) take the initiative in converting manufacturing capacity in response to impact, and (3) strategically recycle and reuse excess manufacturing capacity afterwards in a cost-efficient and orderly manner.

The rest of the paper is organized in four sections. The proposed framework is elaborated in Section 2, followed by the enabling technologies in Section 3. The implementations of Industrial Internet-enabled resilient manufacturing in China were briefly recapped in Section 4. Section 5 concludes the paper with open discussions.

## A Conceptual Framework for Resilient Manufacturing Strategy

**Figure 1:**
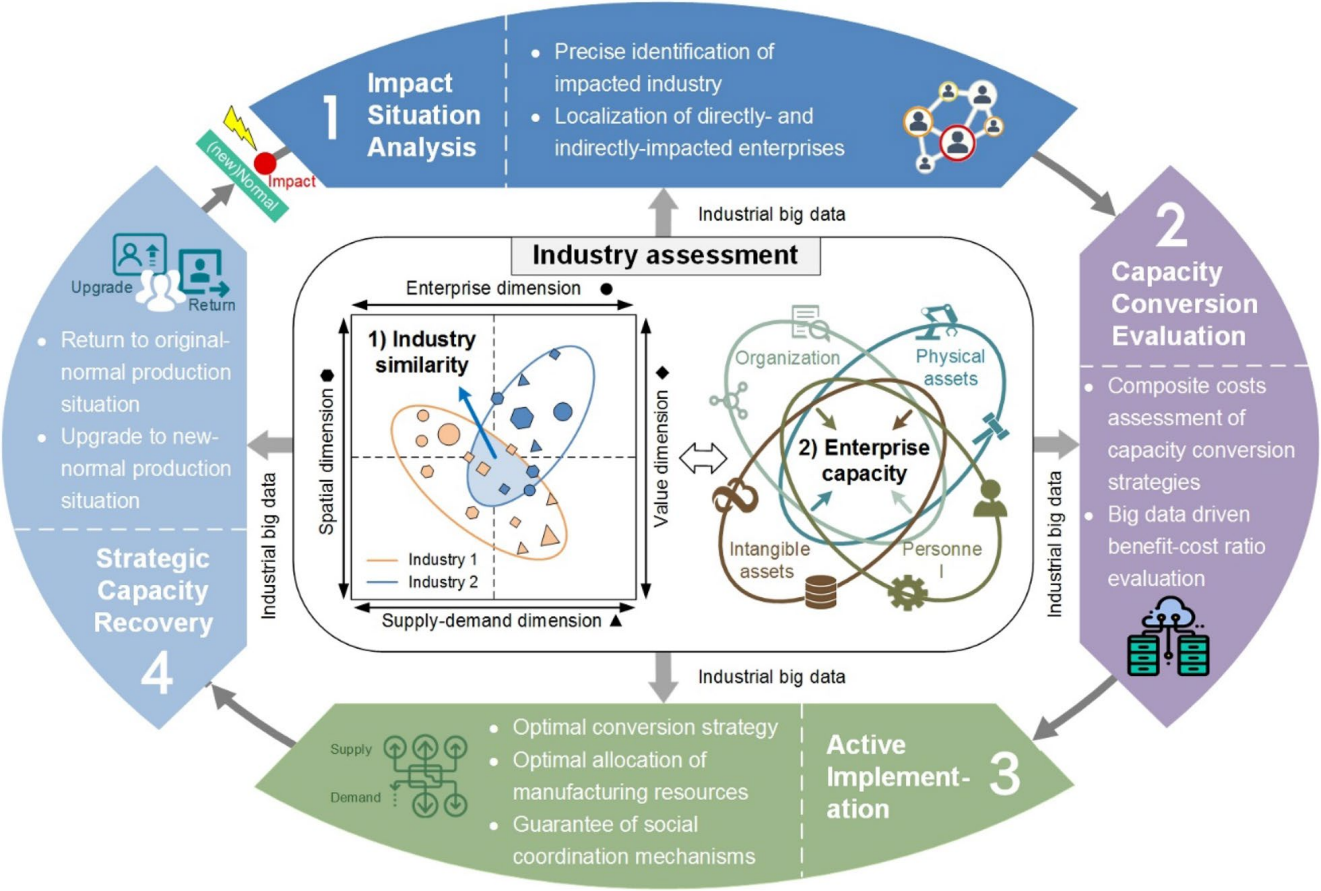
A conceptual framework for resilient manufacturing strategy

The proposed framework consists of two parts, a core and a closed-loop process. The former is represented by “Industry assessment” supporting the four-phase, closed-loop process of resilient manufacturing strategy (see Figure 1). Industry assessment aims to evaluate the feasibility and ability of an enterprise to achieve cross-industry capacity conversion. Such an evaluation of feasibility closely relates to its industrial characteristics and actual capability. This is the core and main driver, which provides the industrial data-information-knowledge-wisdom (DIKW) support for all four phases.

Industry similarity can be first analyzed from four dimensions, that is, spatial dimension (e.g., regional industry cluster, resource distribution), enterprise dimension (e.g., scale, qualification), value dimension (e.g., marketization, foreign trade dependency, talent structure), and supply-demand dimension (e.g., production factors, supply-demand relationship). Following this analytical approach, an enterprise can evaluate its capacity based on the data collected in four categories, organization, physical assets, intangible assets and personnel. On the occasion of a significant supply-demand imbalance or uncertainty, the four-phase, closed-loop resilient manufacturing strategy will be triggered and supported by such a core. Eventually, the original normal or a stable new-normal situation will be achieved. Four phases are described as follows.

### Impact Situation Analysis

When a severe incident/event happens, instant supply shortage, pervasive logistics disruption and abrupt change of demand will lead to supply-demand imbalance. In the first phase, the precise identification of impacted industry and localization of directly- and indirectly-impacted enterprises will be performed. Model-based scenario design and simulation, considering impact propagation mechanism, e.g. ripple effect, can be performed with the data support from the industry assessment core. For example, actual production and dynamic market data can be used to calculate the gap between available capacity and market demand, quantify the capacity deficiency and prioritize targets.

### Capacity Conversion Evaluation

Although targeted industries or even products are known, rush to the rescue may not be a wise and sustainable choice. For an individual enterprise, a comprehensive but prompt evaluation on its capacity conversion feasibility based on, once again, the industrial assessment core is essential. It is required to assess the composite costs, including capital, material, machine, technology and labor costs, of different capacity conversion strategies. Resilient manufacturing strategy enables proactive response to impacts, which means even in the course of capacity conversion, the number and type of machine remains adaptable to the dynamic needs. A big data-driven benefit-cost ratio evaluation for capacity conversion is suggested to improve cost-effectiveness at a national level.

### Active Implementation

As aforementioned, resilient manufacturing covers the ability to take the initiative and responsive in converting manufacturing capacity. This, in most cases, involves the reconfiguration and reconstruction of a manufacturing system. In the third phase, three tasks will be executed collaboratively and rapidly based on industrial big data by a number of enterprises. Task 1, an optimal conversion strategy is finalized with the consideration of overlapping and complementary capacities among different enterprises. Task 2, an optimal allocation of manufacturing resources is determined to compensate the supply-demand imbalance by integrating data and resources among different enterprises. Task 3, a social synergetic mechanism, covering incentive mechanism, cooperative mechanism, competitive mechanism, and etc., is simultaneously established to facilitate the interests of involved enterprises.

### Strategic Capacity Recycle

To reflect the resilient manufacturing strategy, an enterprise should be able to efficiently return to its original-normal situation or stably operate at its new-normal situation after the impact. In addition, recycle and reuse excess manufacturing capacity should be studied for sustainability. A data-driven supply-demand relationship in the post-impact phase should be carefully portrayed with dynamic capacity-demand data. This greatly assists the decision-making process for excess capacity management.

Two possible disposals are (1) recycle excess capacity and return to original-normal production situation, including reconfigure production lines, recycle materials, recover capital investment; (2) reuse excess capacity to transform and upgrade to new-normal production situation, including redesign product/service, reinforce supply chain, rediscover market needs.

## Enabling Technologies for Resilient Manufacturing Strategy

Resilient manufacturing strategy is characterized by agile response and organized recovery, which depends on the comprehensive accurate real-time data from enterprise, as well as the flexible resources organization. Industrial Internet combines big data analytics, cloud computing, artificial intelligence (AI) and other emerging technologies [8]. The capability of big data aquisition, analysis, and intelligent service via cloud-based platforms lays the foundation of ubiquitous connection, resilient supply, and dynamic optimization of manufacturing resources.

In addition, 5G, 3D printing, extended reality (XR), and etc., also provide supporting technologies for the resilient manufacturing. Related technologies are shown in Table 1.

**Table 1:** Enabling technologies for the four phases in resilient manufacturing strategy

Technologies	Phases			
	Impact situation analysis	Capacity conversion evaluation	Active implementation	Strategic capacity recycle
Data acquisition management	√	–	√	√
Big data analysis	√	√	√	√
Intelligent service	–	√	√	√
5G	√	–	√	√
3D printing	–	–	√	–
XR	–	–	√	–

### Data Acquisition and Management

Industrial Internet acquires industrial data in depth cross a large data span based on ubiquitous sensing and cross-domain interaction, including sensing the production terminal data from processing equipment, industrial robots within each enterprise, tracking daily activity data from process, quality, energy consumption, and personnel, and collecting external data from market demand, logistics, and business environment.

Ubiquitous data acquisition provides accurate data for comprehensive capacity evaluation and industry similarity assessment, which supports quantitative analysis on potential cross-industry production capacity in the proposed resilient manufacturing strategy. It also facilitates scenario modeling and optimal strategy decision.

### Big Data Analysis

For the proposed framework, big data analysis enables holistic and deep mining of the data streamed via Industrial Internet platforms. It can be used to forecast the upstream demand, monitor the downstream supply, and dynamically quantify capacity deficiency and prioritize targets. On one hand, big data analytics improves the responsiveness of a manufacturing enterprise, for example, effective suggestions can be given on predictive maintenance and production capacity monitoring. On the other hand, an Industrial Internet platform is conducive to upstream and downstream resource and data integration in the industrial chain, so that to analytically estimate market demand, and manufacturing and logistics performance. Big data analysis can also be used in evaluating the unexpected impact on supply-demand disruptions and identifying the most serious crux [9].

### Intelligent Service

In intelligent services, an industrial Internet platform plays two critical roles. (1) It assists enterprises by integrated control of design, develop, supply and manufacturing. The optimized configuration of manpower, machines and materials, and timely response to demand shift can be achieved. (2) It rapidly allocates cross-enterprise resources. Each enterprise profiles its manufacturing resource on an Industrial Internet platform, which provides data support for industrial AI-driven resources coordination. Existing cloud-based intelligent services can be found as cloud design and development capability, advanced production scheduling, industrial AI engines, and many more.

### Others Related

As the rapid transformation of a manufacturing enterprise puts forward higher requirements on workforce capability, the training, education and operational guidance for front-line workforce are critical. XR technologies can reduce the the workload and cognitive pressure of workers and shorten the training cycle [10]. For example, providing a visualized training, such as Thingworx plus augmented reality (AR), for equipment maintenance, enhancing human-machine interaction with wearable devices, and displaying standard operating procedure (SOP) guidance interactively.

Advanced manufacturing technologies such as 3D printing and industrial robotics can enhance the technological foundation for flexible and intelligent manufacturing systems. In addition, the application of 5G boosts Industrial Internet by breaking through data transmission bottlenecks, e.g. delay, reliability. This further improves the responsiveness and decision-making efficiency in the proposed resilient manufacturing strategy against unexpected impacts.

## China’s Implementation of Industrial Internet-Enabled Resilient Manufacturing

Industrial Internet has played an important and effective role in the prevention and control of the pandemic and the production resumption in China. Ministry of Industry and Information Technology of China therefore vigorously selected the first batch of 66 advanced and instructive Industrial Internet-enabled solutions [11], and grouped as 3Rs according to the characteristics, “manufacturer capacity Recovery”, “supply chain Resilience” and “emergency Response”.

**Figure 2:**
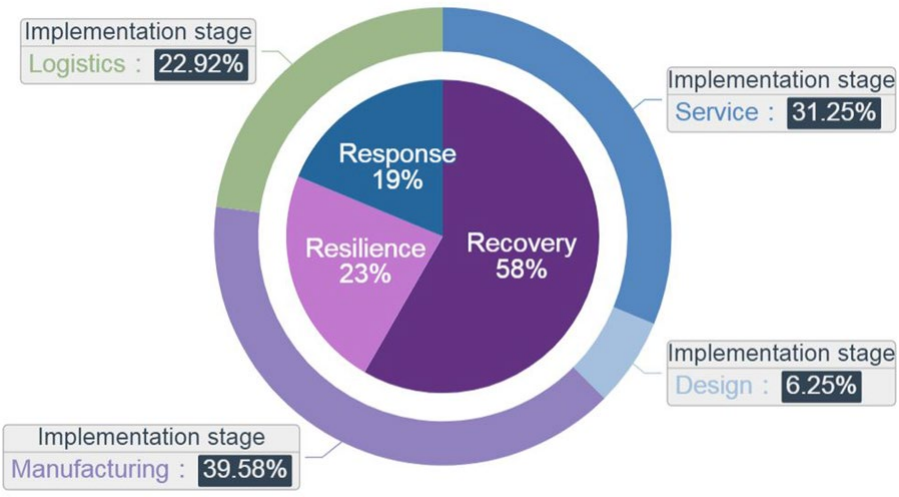
A pie chart for industrial cases of 3Rs

There are 48 industrial cases related to resilient manufacturing framework, and the proportion of each case is shown in Figure 2. By summarizing the practice of resuming production in response to emergencies, China's Industrial Internet platform covers manufacturing domains such as industrial equipment, shipbuilding, machinery, and medical supplies, and carries out practices in aspects of capacity monitoring, supply-demand information exchange, remote operation and maintenance, resource sharing, and collaborative industrial chain.

**Figure 3:**
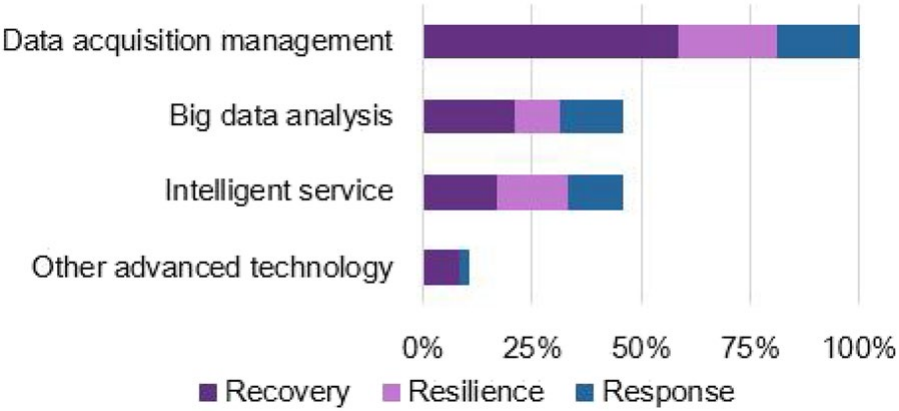
A technological histogram for industrial cases of 3Rs

As outlined in Section 3, the data acquisition and management techniques were applied to all cases. Big data analysis technology and intelligent service technology were utilized by 50% of the cases. Other technologies, such as 5G and AR, were also applied in a few cases (see Figure 3).

There were 28 cases of “manufacturer capacity recovery”, covering research and development, design, production management, operation and maintenance services, and use the data acquisition technology of cloud platform to collect real-time data to digitized personnel, equipment, process, materials and so forth. Big data analysis enables requirements of capacity monitoring (ASUN Industrial Internet Platform), process traceability and predictive analysis, and promotes the sharing of manufacturing capacity and resources.

The developed intelligent methodologies provided remote cloud collaborative design and development (PERA Global), PHM (XSON industrial Internet platform), collaborative manufacturing (ISESOL industrial Internet platform) and other services. Among them, the solutions of 3 companies integrated Industrial Internet and 5G technology, and 1 company used AR technology (Aveo Intelligent Cloud Company). 11 cases of “supply chain resilience” mainly addressed the problem of industrial chain collaboration at a supply chain level, and a few linked smart factories and supply chain (COSMOplat, CASICloud). In the process of deconstruction and reconstruction of supply chain, big data was used to monitor product and supply logistics, track and predict shipping orders, in order to secure supply chain.

Intelligent matching services provided precise matching between supply and demand from upstream to downstream, as well as information sharing and collaboration. For example, the transformation enabled by Haier COSMOplat took 48 hours to build the first automatic production line of medical masks in Shanxi. 9 cases in “emergency response” relied on big data analysis to respond to the urgent demand by 24-hour real-time supervision on production capacity. They allowed the government and enterprises to dynamically perceive and predict the changing trend of production resumption.

Industrial Internet can also realize the integrated management and control of cloud and enterprise, resource scheduling optimization and accurate decision-making, e.g., supOS Industrial Internet platform, via platform docking, so as to improve the response speed to the impact.

## Discussions and Conclusions

In both pandemic preventive control and manufacturing resumption, Industrial Internet has played powerful and effective roles in accurate supply-demand balancing, 24-h online collaborating, and optimized resource sharing and allocating. These roles opportunely enable resilient manufacturing strategy. Nevertheless, existing Industrial Internet-based solutions are rare for proactive assessment and comprehensive evaluation, particularly with the integration of 5G, AI and other emerging technologies. The use of cross-industry data aggregation, fusion and value-mining needs to go deeper. Besides, current implementations focus on emergency supply and production, and seldom perform cross-industry capacity evaluation and resource allocation. Strategic and cost-efficient capacity recycle remains untouched.

This paper provides preliminary reflections and discussions on resilient manufacturing strategy in the wake of COVID-19 pandemic. However, it is worth noting that not every manufacturing enterprise should be resilient at a same high level. It depends on an industry-specific and comprehensive selection, which should be coordinated with the guiding policy and supporting regulations at the national, at least regional level.
